# Montreal Cognitive Assessment test: Psychometric analysis of a South African workplace sample

**DOI:** 10.4102/ajopa.v6i0.151

**Published:** 2024-02-13

**Authors:** Charles H. van Wijk, Willem A.J. Meintjes, Chris J.B. Muller

**Affiliations:** 1 Department of Global Health, Faculty of Medicine and Health Sciences, Stellenbosch University, Cape Town, South Africa; 2 Institute for Maritime Medicine, Simon’s Town, South Africa; 3 Department of Statistics and Actuarial Science, Stellenbosch University, Stellenbosch, South Africa

**Keywords:** cognition, dimensionality, grey-zone thresholds, language, measurement invariance, screening, validity

## Abstract

The Montreal Cognitive Assessment (MoCA) test is a widely used tool to screen for mild neurocognitive impairment. However, its structural validity has not been fully described in South Africa. The study aimed to replicate and extend earlier work with South African samples, to provide an expanded description of the psychometric properties of the MoCA. The study examined the MoCA in a sample of neurocognitively healthy working adults (*N* = 402) and individuals diagnosed with mild neurocognitive disorders (*N* = 42); both groups reported good English proficiency. Analysis included general scale descriptions, and structural and discriminant validity. Age and language, but not gender, influenced MoCA scores, with mean total scores of healthy individuals falling below the universal cut-off. Structural analysis showed that a multidimensional model with a higher-order general factor fit the data well, and measurement invariance for gender and language was confirmed. Discriminant validity was supported, and receiver operating characteristics curve analysis illustrated the potential for grey-zone lower and upper thresholds to identify risk.

**Contribution:**

This study replicated previous findings on the effects of age, language and gender, and challenged the universal application of ≤ 26 as cut-off for cognitive impairment indiscriminately across groups or contexts. It emphasised the need for context-specific adaptation in cognitive assessments, especially for non-English first language speakers, to enhance practical utility. Novel to this study, it extended knowledge on the structural validity of the test and introduced grey-zone scores as a potential guide to the identification of risk in resource-restricted settings.

## Background

### Introduction

The current worldwide prevalence of dementia is expected to double every 20 years, with two-thirds of people with dementia living in developing countries (Potocnik, 2013). Estimates in rural South African communities reach 12%, considerably higher than the worldwide estimate of 4% (De Jager et al., 2017). The sharpest increase in prevalence is expected to occur in low- and middle-income countries, where healthcare services continue to operate under clinical and human resource constraints.

Mild cognitive impairment (MCI) represents an intermediate state between normal cognition and dementia, and reflects a ‘transitional condition between the cognitive changes typically associated with normal ageing and those changes that meet the criteria for dementia’ (APA, 2023a), and precedes and leads to dementia in many cases (Nasreddine et al., 2005). Mild cognitive impairment is associated not only with advancing age but also with other medical conditions.

The need for inexpensive, brief and reliable screening tools in resource-constrained contexts is widely accepted. When advanced sophisticated scanning or neuropsychological assessment is not readily available – as is typical in primary healthcare facilities – reliance on neurocognitive screeners to guide clinical decision-making becomes important.

One popular screener is the *Montreal Cognitive Assessment test*, commonly referred to as the MoCA, which was developed as a brief screening tool with high sensitivity and specificity for detecting MCI (Nasreddine et al., 2005).

### Montreal Cognitive Assessment test

The MoCA is typically used as a broad screen for *global neurocognitive functioning* over multiple domains, where lower scores would suggest neurocognitive difficulties. The test consists of a number of tasks, and the total score reflects performance across six cognitive domains, namely visuospatial, executive, attention, language, memory and orientation. The task contribution to the domains can be seen in Table 1.

**TABLE 1:** Montreal Cognitive Assessment items per domain and correct responses per item.

Domain	Subset items ‘tasks’	Max score possible	Sample		Correct responses	
			Mean	s.d.	*n*	%
**Visuospatial**		4	3.33	0.75	-	-
	Cube copy	-	0.69	0.46	279	69.4
	Clock contour	-	0.96	0.19	387	96.3
	Clock numbers	-	0.87	0.34	349	86.8
	Clock hands	-	0.81	0.40	324	80.6
**Executive**		3	2.32	0.69	-	-
	Trail making	-	0.84	0.37	336	83.6
	Bicycle-train	-	0.92	0.27	370	92.0
	Watch-ruler	-	0.57	0.50	228	56.7
**Attention**		6	5.31	0.85	-	-
	Digits forwards	-	0.87	0.34	350	87.1
	Digits backwards	-	0.86	0.35	344	85.6
	Vigilance/tapping	-	1.00	0.00	401	99.8
	Serial 7’s 4–5 correct	-	-	-	267	66.4
	Serial 7’s 2–3 correct	-	-	-	108	26.9
	Serial 7’s 1 correct	-	-	-	24	6.0
	Serial 7’s 0 correct	-	-	-	3	0.7
	Serial 7 total	-	2.59	0.64	-	-
**Language**		6	4.66	0.97	-	-
	Lion	-	1.00	0.00	402	100.0
	Rhinoceros	-	0.94	0.27	376	93.5
	Camel	-	0.96	0.21	384	95.5
	Sentence 1 (repeat)	-	0.74	0.44	296	73.6
	Sentence 2 (repeat)	-	0.30	0.46	122	30.3
	Fluency	-	0.73	0.45	293	72.9
**Memory**		5	3.86	1.09	-	-
	First trial	-	4.58	0.61	-	-
	Second trial	-	4.90	0.035	-	-
(delayed recall)	Face	-	0.69	0.46	276	68.7
	Velvet	-	0.78	0.42	313	77.9
	Church	-	0.86	0.35	345	85.8
	Daisy	-	0.66	0.48	265	65.9
	Red	-	0.87	0.33	351	87.3
**Orientation**		6	6.00	0.00	-	-
	Date	-	1.00	0.00	402	100.0
	Month	-	1.00	0.00	402	100.0
	Year	-	1.00	0.00	402	100.0
	Day of week	-	1.00	0.00	402	100.0
	Place	-	1.00	0.00	402	100.0
	City	-	1.00	0.00	402	100.0

**Total**		**30**	**25.46**	**2.37**	**-**	**-**

s.d., standard deviation.

The maximum obtainable score is 30, and if a patient has 12 years or less of education, the total score is corrected by adding one point. A total score of ≤ 26 was traditionally considered as universally indicative of MCI and would warrant referral for further investigation and management. The original validation of the test, in English- and French-speaking Canadians, reported a sensitivity of 90% and a specificity of 87% for detecting MCI (Nasreddine et al., 2005).

While the MoCA is generally used to screen for neurocognitive disorders associated with advancing age, for example, dementia (Chou et al., 2014; Freitas et al., 2012a; Hoops et al., 2009), it has also shown promise for use in other settings, for example, sepsis survivors (Brown et al., 2018), and patients with brain metastases (Olson et al., 2008) or transient ischaemic attacks (Pendlebury et al., 2010). More recently, studies suggested that the MoCA could be sensitive enough to detect cognitive impairments (across various domains) in patients with a history of coronavirus disease 2019 (COVID-19) (Crivelli et al., 2022).

Although the MoCA was originally developed for use with North American adults at risk of developing Alzheimer’s disease, it has since been validated, translated and adapted across multiple countries, languages and cultures, including Brazilian, Korean, Japanese and Arabic versions (Fujiwara et al., 2010; Lee et al., 2008; Pinto et al., 2019; Rahman & El Gaafary, 2009). Translation into Southern African languages includes Kiswahili, Afrikaans and isiXhosa (Masika et al., 2021; Rademeyer & Joubert, 2016; Robbins et al., 2013). Different thresholds indicative of MCI have been recommended in different contexts (e.g. Freitas et al., 2013; Masika et al., 2021; Thomann et al., 2020). Furthermore, to maintain validity in the context of MCI, the scores of screening instruments should not be influenced by a patient’s language, cultural background or level of education (Ng et al., 2018; Wilder et al., 1995), which has generated interest in the cultural and language appropriateness of MoCA items for people across different cultural-linguistic backgrounds.

### The South African experience with the Montreal Cognitive Assessment

A number of local South African studies used the MoCA to investigate a range of conditions and contexts. An overview is briefly presented in Table 2. In summary (Beath et al., 2018; Kirkbride et al., 2022; Mienie, 2020; Robbins et al., 2013), the total mean scores for cognitively healthy groups were consistently below the established cut-off score for MCI, thus inaccurately identifying people as MCI even though they were cognitively healthy. Floor and ceiling effects were regularly reported, and indications of cultural bias, independent of level of education, were observed. Total scores correlated with age and education, but not gender, and varying outcomes on validity were reported, depending on type (e.g. criterion vs. discriminant, etc.). There was thus consensus that the MoCA would need to be modified in order to differentiate between normal ageing and MCI in the South African population. As a result, there were regular calls to abandon the universal cut-off point of 26, particularly in heterogeneous samples, with different thresholds recommended for different contexts, such as lowering the threshold to ≤ 24 for local use.

**TABLE 2:** Summary of Montreal Cognitive Assessment studies with South African samples.

Source and aim	Method	Relevant findings	Authors’ conclusions
Robbins et al. (2013) Explored utility of MoCA to detect HIV-associated neurocognitive disorder	HIV-positive and matched controls *N* = 39 for each group Used isiXhosa version	Mean total for healthy controls = 21.7 (± 2.0) Floor effects for: cube drawing rhinoceros naming serial 7 abstraction tasks	MoCA would need modification before it can be validated and normed for use with South African population.
Beath et al. (2018) Explored validity and effectiveness of MoCA to screen for MCI	Cognitively healthy adults *N* = 370 Used English version	Cronbach’s α = 0.624 Means scores significantly (*p* > 0.001) correlated to gender (*r* = −0.199), age (*r* = −0.203) and education (*r* = 0.326) Strong correlation with the RBANS (*r* = 513; *p <* 0.001), suggesting good criterion-related validity MoCA for predicting MCI (using ROC) was fair with AUC = 0.79 Using original cut-off score of 26: sensitivity = 94.2% specificity = 28.2% When cut-off score = 23: sensitivity = 75% specificity = 66.7%	MoCA appears *fairly reliable* at identifying MCI in this population, but that some modification to certain domains and items are needed to improve differentiation between normal ageing and MCI. Suggest lowering cut-off score to 24, until such time that culturally adapted version of MoCA has been developed and validated.
Mienie (2020) Explored appropriateness of MoCA as culturally sensitive screening test for cognitive impairment in Sesotho-speaking population	Cognitively healthy adult Sesotho-speaking healthcare users *N* = 93 Used English version	Cut-off score of 26 yielded false-positive diagnosis of MCI for 67.7% of sample Total mean score for: sample with secondary level education = 23.9 for sample with tertiary level education = 25.0 Reported indicators of cultural bias, independent of level of education, seen in floor effects for: cube drawing fluency serial 7 second repeat sentence delayed recall of ‘daisy’ trail making vigilance rhinoceros naming	Because of language, educational or cultural bias, original English version of MoCA not appropriate screening tool for MCI in study.
Kirkbride et al. (2022) Explored impact of demographic variables, internal consistency and discriminant validity	Control sample: Healthy South African adults with English as second or third language, educated in public schools *N* = 89 Clinical sample: HIV-positive patients with psychiatric or neurocognitive comorbid disorder *N* = 83 Used English version	Cronbach’s α = 0.64 Total mean scores significantly correlated with years of education (*r* = 0.38) and age (*r* = −0.28) but not gender Poor discriminant validity	Need to abandon universal cut-off point, particularly in heterogeneous samples.

MoCA, Montreal Cognitive Assessment; HIV, human immunodeficiency virus; MCI, mild cognitive impairment; RBANS, repeatable battery for the assessment of neuropsychological status; ROC, receiver operating/operator characteristics; AUC, area under the curve.

### Summary of psychometric findings

#### Scale structure (dimensionality)

Analysis of the factorial structure of the MoCA may lead to three outcomes (Sala et al., 2020, pp. 155–156). Firstly, it may indicate the presence of one latent general factor (i.e. unidimensionality), where the test is measuring the one construct of interest with some reliability. Secondly, there may be more than one latent factor (i.e. multidimensionality), but without a general factor. In such a case, the total test score is not particularly meaningful because it does not refer to any general construct. Thirdly, the factorial structure may be both multidimensional *and* all the test items correlate with each other. This would suggest that the total test score measures a presumed general factor.

Some studies reported substantially unidimensional structures (Freitas et al., 2015; Luo et al., 2020). Other studies found the MoCA to be multidimensional with no general factor, although the number of factors were unclear (Coen et al., 2016, Duro et al., 2010). Other researchers reported the tendency of the items of the MoCA to converge towards a multidimensional structure with a general factor (Freitas et al., 2012b). Different findings appear to reflect different methodologies. Earlier confirmatory factor analyses (CFA) have been criticised for using suboptimal techniques in dealing with binary data (Sala et al., 2020). South African studies either did not conduct structural analysis or did not report the specific techniques they employed.

Recent studies, using well-described CFA techniques, reported the presence of a general factor with multiple subfactors, suggesting that the total score is indeed a measure of global cognitive functioning (Sala et al., 2020). This corroborated the earlier assumption of a general factor (Freitas et al., 2015; Luo et al., 2020), with several subfactors (Sala et al., 2020).

#### Measurement invariance

Measurement invariance is an important property of a test, as it indicates whether responses to items have the same meaning under different conditions (e.g. in different gender or language groups). Without establishing measurement invariance, it is difficult to make meaningful comparisons across groups. Only one MoCA study could be located (Sala et al., 2020) that confirmed measurement invariance for age, gender, education and economic status, in a large sample of older Japanese participants.

#### Internal consistency reliability

Previous studies found acceptable to adequate internal consistency (cf. Sala et al., 2020, for summary), with Cronbach’s α of 0.62–0.64 for South African samples reported (Beath et al., 2018; Kirkbride et al., 2022). However, Cronbach’s α (representing a total factor saturation index) is not necessarily trustworthy when the assumption of unidimensionality is not met (Reise et al., 2013), and an index of general factor saturation such as McDonald’s ω (Dunn et al., 2014) is more appropriate. South African reports on internal consistency exclusively described Cronbach’s α, which is a limitation, given both the categorical nature of MoCA item scores and the absence of structural analysis in those studies.

#### Sociodemographic variables

Decreasing scores with advancing age have consistently been reported (Elkana et al., 2020; Malek-Ahmadi et al., 2015; Pinto et al., 2018), also in South African samples (*r* = −0.20 to −0.28; Beath et al., 2018; Kirkbride et al., 2022). Some studies reported significant differences in female and male performance (Lu et al., 2011), whereas others did not (Robbins et al., 2013; Santangelo et al., 2015). Recent South African reports are conflicting, indicating either significant gender effects (Beath et al., 2018) or absence of any significant gender difference (Kirkbride et al., 2022). Differences in sample demographics may contribute to such inconsistency.

#### Language of administration

The impact of language on test performance is well understood in South Africa’s multilingual population (Ferrett et al., 2014; Watts & Shuttleworth-Edwards, 2016). South African studies do not always report language (of participants or of administration), but those that did also reported poor outcomes when the English version was administered to respondents who were not native English speakers, and they expressed concern about the validity of the MoCA as a screening or diagnostic tool (Kirkbride et al., 2022; Mienie, 2020).

#### Discriminant validity

After the original validation of the test showed acceptable sensitivity and specificity for detecting MCI (Nasreddine et al., 2005), numerous validation studies – from different regions and languages – subsequently also reported fair sensitivity and specificity (e.g. Fujiwara et al., 2010; Gil et al., 2015; Nasreddine & Patel, 2016; Ozdilek & Kenangil, 2014; Yeung et al., 2014). As mentioned, South African data were less supportive of its ability to discriminate between healthy adults and cognitive impairment, with authors consistently concluding that modification may be required to reliably identify MCI (Beath et al., 2018; Kirkbride et al., 2022; Mienie, 2020; Robbins et al., 2013).

There is a further concern with the use of absolute cut-off points. Neurocognitive performance is vulnerable to intrapersonal and situational factors on the day of administration, as well as to the human fallibility of the administrator. A single cut-off point may also be inadequate to discriminate between persons with possible MCI and those without. One solution is to determine a so-called grey zone, with a lower limit cut-off to support sensitivity (the ‘at-risk’ threshold – interpreted as requiring closer surveillance) and an upper limit cut-off to support specificity (the ‘intervention’ threshold – interpreted as requiring action).

### Aim and objectives

This was a replication and extension study, building on earlier work done with South African samples. It aimed to provide an expanded description of the psychometric properties of the MoCA in a group of neurocognitively healthy (NCH) working adults who reported good English proficiency. This was to be done through three objectives, namely:

- Objective 1: To replicate local studies that provided general scale descriptions, including total and domain scores, and sociodemographic considerations, particularly those of age, gender and home language.
- Objective 2: To extend psychometric analysis to consider structural validity (including dimensionality, internal consistencies and measurement invariance), based on the framework of Sala et al. (2020).
- Objective 3: To replicate local studies that provided indications of discriminant validity, by differentiating between NCH individuals and a sample with diagnosed mild neurocognitive disorders (MND), and examining the MoCA’s usefulness to identify at-risk individuals. This will be extended by illustrating the usefulness of developing grey-zone lower and upper thresholds.

## Methods

### Overview

This study entailed a retrospective review of clinical records, obtained from two archives. Data for the NCH sample – used for structural validity analysis – were sourced from the records of an occupational health surveillance programme that included workers in full-time employment across a range of occupational fields. Depending on occupational field and workplace characteristics, the programme included a baseline MoCA administration, archived for later reference. The study employed quota sampling to enable reasonably equal distribution across age and gender categories (APA, 2023b). Individual cases were successively included until each age-by-gender subsample was saturated. Data for a clinical sample were sourced from the records of a neuropsychological clinic, with a multidisciplinary team diagnosis of MND. Data were collected during 2020–2022.

### Participants

Inclusion criteria for the NCH group were age 20–60 years, with a grade 12 or higher level of education, and a self-reported proficiency in English. Exclusion criteria were any known pre-existing cognitive disorders, head injury or physical illnesses that would better explain neurocognitive health status. Furthermore, no acutely ill patients (at the time of MoCA administration) were enrolled in the study.

The sample of 402 participants consisted of 196 (48.8%) women and 206 (51.2%) men. All participants were in possession of grade 12 plus vocational training, which consisted of either national diplomas or 2- or 3-year vocational training certificates. They were all considered highly skilled workers and represented a wide range of vocational backgrounds, including technical/engineering (25.4%), clerical/administrative (21.2%), security (17.2%), catering/hospitality (12.2%) and radar/sonar operators (11.0%). The sample does not necessarily represent any larger community or industry in South Africa. Official workplace language was reported as English, and all participants self-identified as proficient in English. Distribution of reported home language was as follows: English 126 (31.3%), Afrikaans 73 (18.2%), Setswana 50 (12.4%), IsiXhosa 40 (10%), IsiZulu 32 (8.0%), Sesotho 31 (7.7%), Sepedi 20 (5%), Tshivenda 12 (3%), Siswati 9 (2.2%), Xitsonga 5 (1.2%) and Ndebele 4 (1%).

The MND sample consisted of 42 participants, of which 20 (48%) were women and 22 (52%) were men. All had at least 12 years of schooling, but no further educational history was available. Ages ranged from 55 to 60 years. Language preference was reported as English. This was a convenience sample, and cases were included where sufficient data were available (i.e. MoCA total and domain scores, age, gender and home language), and permission to use data for research was available on file.

### Measures and variables

#### Montreal Cognitive Assessment

The NCH sample MoCA was administered in its standard version 7.1 format, in English, by two clinical psychologists experienced in neurocognitive screening. Administrations were randomly allocated to them, based on availability. The English language proficiency of the participants was not objectively assessed. This reflected current practices in the clinical setting.

#### Sociodemographic data

The following sociodemographic data – previously reported to be relevant to MoCA outcomes – were sourced from the archived records: age, gender and home language. Occupational fields were noted for the purpose of sample description only.

#### Brief mental health screeners

On the same day of MoCA administration, participants also completed a brief screen of general mental health, which included the Patient Health Questionnaire for Depression (PHQ-9; Gilbody et al., 2007) and the Generalised Anxiety Disorder scale (GAD-7; Löwe et al., 2008). The screen indicated no cases of concern, and neither were its scores associated with MoCA performance, and it was thus not included in any further analyses.

### Data management and analysis

Statistical Package for Social Sciences (IBM SPSS for Windows, version 27) was used for general statistical analyses, while structural analyses were conducted in R version 4.3.1 (R Core Team, 2023), where CFA models were fitted using the package lavaan (v06-16), and McDonald’s ω and its confidence intervals were calculated using the package MBESS (v4.9.2).

Following Freitas et al. (2012b) and Sala et al. (2020), the 31 dichotomous items of the test were used in the analysis. Scale descriptions included the calculation of means, standard deviations and total score range, as well as the breakdown of task and domain scores. One particular task, namely phonetic fluency, received additional analyses, to explore the influence of home language (i.e. English vs. non-English) on word generation.

The effects of sociodemographic variables were explored using Pearson’s correlation coefficients for age and analysis of variance (ANOVA) – coded here into four groups (20–29, 30–39, 40–49 and 50–60). A *t*-test for independent samples was used to explore gender effect. A *t*-test was also used for language, which was coded into two groups, namely English as first language (31.3%) and not English as first language (68.7%), as well as ANOVA for individual language groups. The effect of different test administrators was explored through a *t*-test for independent samples.

To assess item/domain discriminating power, Pearson’s correlation coefficient was calculated between each item and the total score, between each item and cognitive domain total, and between each cognitive domain and the MoCA total score. Nonsignificant correlation coefficients would indicate the lack of factorial validity, while significant correlation coefficients would be an indicator of factorial validity.

Structural validity was further examined through considering dimensionality, internal consistencies and measurement invariance. Items with a mean correct response rate above 98% were excluded from this analysis to avoid estimation problems related to ceiling effects (Sala et al., 2020). This led to the exclusion of eight items (vigilance, lion naming and the six orientation items). Analyses were subsequently conducted with 23 dichotomous items.

Dimensionality was examined through CFA, which is used to test whether the data fit a hypothesised measurement model. Confirmatory factor analysis was thus conducted to test the previously confirmed multidimensional model, with 23 items loading on five latent factors that all correlate to a higher-order general factor (cf. Sala et al., 2020). Due to the dichotomous nature of the data, WLSMV (weighted least squares means and variance) estimation was used. This model did not allow for meaningful measurement invariance testing, and thus a second model, using the five-factor totals loading onto a higher-order general factor, was also tested.

For a CFA, the global fit χ^2^ would ideally be small and not significant (and χ^2^/*df* = 2–3), but this is rarely achieved in larger samples, and the following indices with cut-off points were also taken into consideration: a root mean square error of approximation (RMSEA) ≤ 0.05 indicates a close fit, while an RMSEA between 0.05 and 0.08 suggests a reasonable approximate fit. The comparative fit index (CFI) should be > 0.90 and the standardised root mean square residual (SRMR) should be < 0.08 (Kline, 2016).

To overcome the potential drawback of Cronbach’s α, internal consistency was examined with McDonald’s ω, specifically categorical ω with bootstrap confidence intervals (Dunn et al., 2014; Kelley & Pornprasertmanit, 2016).

Measurement invariance, as mentioned earlier, refers to the generalisability element of construct validity (Putnick & Bornstein, 2016) and is assessed when scores need to be compared across groups (e.g. gender, language). Scales need to be invariant with respect to the way in which the latent constructs are formed (configural invariance), and the indicators or items should load similarly on latent factors across the groups (metric invariance). Testing for intercept invariance is called scalar equivalence. Testing for invariance is a hierarchical process and cannot proceed to a next level if model fit for a previous level fails. The requirement for invariance is that the difference in global χ^2^ between hierarchical models is not significant. Measurement invariance for the MoCA was evaluated for gender (women and men) as well as language (English-as-first-language speakers and not-English-as-first-language speakers), using the sample of cognitively healthy adults.

Discriminant validity was investigated by conducting *t*-tests for independent samples to determine whether any difference between NCH and MND adults could be observed. This analysis used the NCH sample aged 55–60 years. The *t*-test was significant, and a receiver operating/operator characteristics (ROC) curve analysis was conducted to investigate the MoCA’s usefulness to identify individuals with MCI. This was done by considering the area under the curve (AUC) and sensitivity and specificity ratios. Lower and upper thresholds for screening of MCI were further illustrated using ROC curve analysis outcomes (cf. Dutheil et al., 2017).

## Results

### General description of Montreal Cognitive Assessment data

**FIGURE 1:**
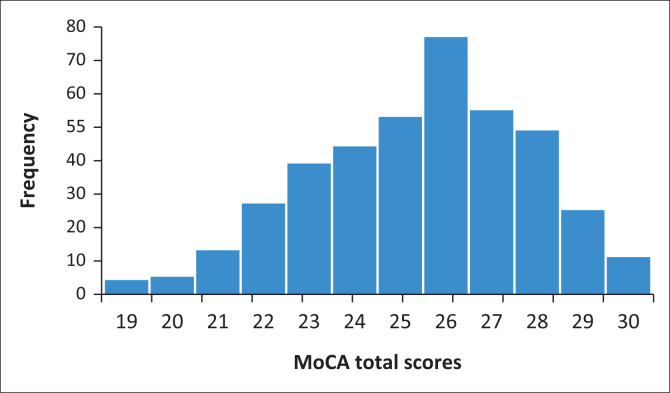
Distribution of Montreal Cognitive Assessment total scores.

Montreal Cognitive Assessment total scores, for the full NCH sample, ranged from 19 to 30, with a mean of 25.46 (± 2.4). The score distribution is visually represented in Figure 1. Task and domain scores can be found in Table 1. In terms of individual item issues, ceiling effects were observed for ‘vigilance’, lion naming and all the ‘orientation’ tasks. Poor performance (compared to the rest of the tasks) was observed for the second abstraction item and the second repeat sentence, while delayed recall of ‘daisy’ was most often omitted.

In 24 cases (of 26) of incorrect naming of the rhinoceros, the words ‘buffalo’ or ‘hippopotamus’ were used. In 12 (of 18) cases where camel could not be named, respondents could describe the animal (e.g. ‘lives in desert’, ‘store water in its back’), even though they failed to name it. Failure to name, in spite of description, was scored as zero.

Word generation (‘phonetic fluency’) totals were also recorded. The use of verbal fluency as proxy for general premorbid ability is controversial (Lezak et al., 2004; Salvadori, 2023), and the actual word counts are included here only to explore possible language effects. The number of words ranged from 3 to 30, with a mean of 12.94 (± 4.0). The number of words produced differed significantly between English-as-first-language speakers and not-English-as-first-language speakers (*t* = 3.048, *p* < 0.01, Cohen’s *d* = 0.34), although the actual difference was only one word (*M* = 13.9 vs. *M* = 12.5, respectively). There was wide variability across individuals within the same general language groups. If the threshold for a positive score on the phonetic fluency item would have been lowered to ≥ 10 (from ≥ 11), then another 7% of the full sample would have scored a point on this item (5% of English-as-first-language speakers and 8% of not-English-as-first-language speakers).

There were no significant differences in the mean total scores of cases distributed between the two psychologists who administered the screener (*t* = 0.533, *p* = 0.127).

### Description of sociodemographic effects

The age-by-gender distribution is presented in Table 3.

**TABLE 3:** Montreal Cognitive Assessment total scores by age and gender subgroups.

Age group	Women			Men			*t*	*p*	Cohen’s *d*	Combined gender	
	*n*	*M*	s.d.	*n*	*M*	s.d.				*M*	s.d.
20–24	25	26.48	1.50	26	25.92	1.83	−1.189	0.120	0.332	26.20	1.69
25–29	26	25.38	2.26	26	26.69	1.87	2.270	0.028*	0.630	26.04	2.16
30–34	26	25.35	3.17	27	26.52	1.99	1.618	0.058	0.445	25.94	2.68
35–39	25	25.44	2.22	27	26.04	2.19	0.975	0.167	0.271	25.75	2.20
40–44	25	26.04	2.15	25	25.48	2.00	−0.953	0.173	0.270	25.76	2.08
45–49	25	24.36	2.25	25	24.92	2.89	0.765	0.224	0.216	24.64	2.58
50–54	25	24.96	2.65	25	24.16	2.01	−1.201	0.118	0.340	24.56	2.37
55–60	19	24.68	2.26	25	24.56	2.68	−0.167	0.434	0.050	24.61	2.48

**Total group**	**196**	**25.36**	**2.40**	**206**	**25.56**	**2.33**	**0.850**	**0.198**	**0.085**	**25.46**	**2.37**

s.d., standard deviation; *M*, mean.

* , *p* < 0.05.

There was a small but significant difference between the total scores of women and men among the 25–29-year-old group (Table 3), but no other significant gender differences per age groups. Furthermore, there was no significant difference between the total scores of women and men (*p* = 0.198) and only a significant difference on one of the domain scores, namely visual-spatial (*t* = 3.187, *p* < 0.01, Cohen’s *d* = 0.36). This is detailed in Table 4. The combined gender groups were used for further analysis of age effects.

**TABLE 4:** Comparison of Montreal Cognitive Assessment total score and domain scores across gender and first language.

Domain	Sample mean	Mean per gender				Mean per language			
		Women	Men	*t*	Cohen’s *d*	English as first language	Not English as first language	*t*	Cohen’s *d*
Visuospatial	3.33	3.19	3.43	3.637**	0.36	3.44	3.28	1.957	0.21
Executive	2.32	2.31	2.32	0.345	0.03	2.34	2.32	0.347	0.04
Attention	5.31	5.22	5.39	2.047	0.21	5.40	5.27	1.585	0.16
Language	4.66	4.70	4.60	−0.804	0.08	5.09	4.46	6.291**	0.68
Memory	3.86	3.94	3.75	−1.591	0.16	3.67	3.94	−2.264*	0.24
Orientation	6.00	6.00	6.00	-	-	6.00	6.00	-	-
Total	25.46	25.37	25.47	0.850	0.09	25.92	25.25	2.645*	0.29
Number of words generated	12.94†	13.08	12.82	−0.519	0.05	13.85	12.53	3.048**	0.34

† , mean ± 4.0, range: 3–30.

* , *p* < 0.05;

** , *p* < 0.01.

There was a significant correlation between age and total MoCA scores (*r* = −0.249, *p* < 0.001), as well as between age and memory (*r* = −0.353, *p* < 0.001). Age correlations with the other five domain totals were not significant.

ANOVA indicated a gradual decline of scores across advancing age (*F*_7,394_ = 4.662, *p* < 0.001), with the difference between highest score (20–24 years) and lowest score (55–60 years) less than 2 points (see Table 3).

There were significant but small differences between the total MoCA scores of the English-as-first-language and not-English-as-first-language groups (*p* < 0.01, Cohen’s *d* = 0.29), as well as on the language (*p* < 0.01, Cohen’s *d* = 0.68) and memory (*p* < 0.05, Cohen’s *d* = 0.24) domain scores. This is also detailed in Table 4.

The mean scores of the 10 South African languages included in the not-English-as-first-language group were also subjected to ANOVA, and no significant differences between the individual 10 languages were found for mean total MoCA scores (*F*_9,266_ = 0.299, *p* = 0.975) or any of the domain scores.

### Structural validity

#### Item-domain-total score correlations

Correlations between individual items and domain totals and total scores are presented in Table 5. Due to a lack of variance, vigilance, lion naming and the six orientation tasks were not included. All items correlated significantly to the total score, except for the contour aspect of the clock drawing task. All item-domain correlations were significant and as expected. A few tasks also correlated (but with small effect size) to domain totals not expected, namely the trailmaking task (executive domain) and Sentence 1 (language domain) that correlated with the attention/working memory domain.

**TABLE 5:** Correlation coefficient of each item with total and domain scores.

Item	Total score	Visual-spatial	Executive	Attention/working memory	Language	Memory
Trailmaking task	0.350**	0.143**	0.472**	0.203**	0.122*	0.108*
Cube copy	0.360**	0.643**	0.124*	0.162*	0.068	0.086
Clock contour	0.027	0.228**	−0.022	−0.035	−0.002	−0.050
Clock numbers	0.160**	0.519**	0.012	0.049	0.015	−0.072
Clock hands	0.268**	0.581**	0.057	−0.019	0.101*	0.068
Naming lion	-	-	-	-	-	-
Naming rhinoceros	0.261**	0.103*	0.168*	0.085	0.358*	0.012
Naming camel	0.256**	0.128*	0.102	0.080	0.322**	0.060
Digits forwards	0.282**	0.022	0.084	0.473**	0.187**	0.017
Digits backwards	0.272**	0.002	0.090	0.510**	0.192**	−0.041
Vigilance	-	-	-	-	-	-
Subtraction 3	0.439**	0.146*	0.089	0.714**	0.147*	0.095
Subtraction 2	−0.296**	−0.119*	−0.065	−0.435**	−0.086	−0.100
Subtraction 1	−0.280**	−0.084	−0.057	−0.525**	−0.118*	0.004
Subtraction 0	−0.115*	0.039	0.001	−0.236**	−0.059	−0.015
Sentence repetition 1	0.370**	0.061	0.027	0.194**	0.578**	0.071
Sentence repetition 2	0.414**	0.070	0.091	0.151*	0.648**	0.098
Phonetic fluency	0.320**	−0.030	0.018	0.179**	0.579**	0.058
Abstraction 1	0.143*	−0.017	0.473**	0.054	−0.009	−0.014
Abstraction 2	0.293**	0.051	0.776**	0.015	0.092	0.018
Recall face	0.302**	0.005	0.045	0.035	0.056	0.546**
Recall velvet	0.352**	0.060	0.042	−0.015	0.147*	0.574**
Recall church	0.218**	0.018	0.025	−0.043	−0.003	0.490**
Recall daisy	0.349**	0.002	0.018	0.105	0.089	0.580**
Recall red	0.239**	0.039	0.049	0.009	0.013	0.451**

Note: Vigilance, lion naming and the six orientation items were not included as there was not sufficient variance in scores.

* , *p* < 0.05;

** , *p* < 0.01.

Correlations between domain and total scores are presented in Table 6. All domain scores correlated significantly and with large effect sizes to total scores. Other interdomain correlations had small effect sizes.

**TABLE 6:** Correlation coefficients of the cognitive domains and total score.

Item	Total score	Visual-spatial	Executive	Attention/working memory	Language	Memory
Visual-spatial	0.445**	-	-	-	-	-
Executive	0.456**	0.107*	-	-	-	-
Attention/working memory	0.574**	0.103*	0.142*	-	-	-
Language	0.635**	0.102	0.129*	0.282**	-	-
Memory	0.558**	0.044	0.066	0.044	0.121*	-

Note: The domain of orientation was not included as there was no variance in scores.

* , *p* < 0.05;

** , *p* < 0.01.

#### Dimensionality

The multidimensional model, with individual items loading on five latent factors, and all correlating to a higher-order general factor, was subjected to a CFA. Although the model did not obtain a nonsignificant χ^2^ (χ^2^ = 215.027, *df* = 165, *p* < 0.01; and CFI = 0.581), the χ^2^ value was not excessively high (and χ^2^/*df* = 1.303), and the RMSEA (0.027; 90% CI: 0.016–0.037) and SRMR (0.054) were adequately small, suggesting an acceptable fit to the data.

A second model, using the five-factor totals with a higher-order general factor, was also tested. Confirmatory factor analyses indicated a close model fit (χ^2^ = 3.086, *df* = 5, *p* = 0.687), supported by low RMSEA (*p* = 0.000; 90%: 0.00–0.53) and SRMR (0.020) and high CFI (1.0). Domains loaded from 0.19 (memory) to 0.53 (language). The results suggest an excellent fit to the data.

#### Internal consistency reliability

McDonald’s categorical ω – calculated using the dichotomous individual items (excluding the eight ceiling items and serial 7s) – was 0.423 (95% CI: 0.029–0.519). McDonald’s ω – using the five domain totals – was 0.399 (95% CI: 0.290–0.577). The McDonald’s ω calculations suggest poor internal consistency.

#### Measurement invariance

The model using the MoCA domain scores showed acceptable configural and metric invariance (Δχ^2^ = 1.094, Δ*df* = 4, *p* = 0.895) for gender, but did not achieve scalar invariance (Δχ^2^ = 20.974, Δ*df* = 4, *p* < 0.001). Similarly, the model showed acceptable configural and metric invariance (Δχ^2^ = 3.933, Δ*df* = 4, *p* = 0.415) for language, but again did not achieve scalar invariance (Δχ^2^ = 16.877, Δ*df*= 4, *p* < 0.01).

### Discriminant validity

Table 7 presents the frequency of total scores from 26 to 21, for the NCH group. When the previously recommended score of ≤ 26 was used as threshold for probable MCI, the MoCA would have – incorrectly – identified 65% of the current NCH sample as suffering from possible cognitive impairment. Even at the locally recommended lowered threshold of ≤ 24, the scale would still incorrectly identify 33% of the sample with possible MCI. While home language played a role here, it did not explain performance fully, for when only the English-as-first-language speakers were counted (at ≤ 26), almost 60% were still identified as at-risk for MCI.

**TABLE 7:** Frequencies of total scores.

Threshold for MCI	Full sample		English as first language (%)	Not English as first language (%)
	*n*	%		
≤ 26	262	65.2	59.5	68.7
≤ 25	185	46.0	41.3	49.6
≤ 24	132	32.8	27.0	37.3
≤ 23	88	21.9	17.5	26.1
≤ 22	49	12.2	7.9	16.5
≤ 21	22	5.5	4.0	8.8

MCI, mild cognitive impairment.

Performance difference between the NCH group (aged 55–60 years) and the MND group was explored with a *t*-test for independent samples. The NCH sample (*M* = 24.61, ± 2.5, range: 19–30) performed significantly better (*t* = 9.392, *p* < 0.001, Cohen’s *d* = 2.0) than the MND sample (*M* = 18.90, ± 3.1, range: 12–24) of similar age.

The ROC curve analysis indicated good probability in predicting cases of MND (AUC = 0.923). Optimal cut-off was at ≤ 22 (sensitivity = 91%, specificity = 76%) or ≤ 23 (sensitivity = 80%, specificity = 86%).

For illustration, the lower and upper thresholds for screening of MCI were determined with a ROC curve analysis (Dutheil et al., 2017). This process identified a score of < 21 as the lower threshold (‘action required’) and a score of < 24 as the upper threshold (‘at risk’).

## Discussion

This replication and extension study built on earlier work with South African samples and used a group of NCH working adults who reported good English proficiency. It set out three objectives.

The first objective was to provide general scale descriptions and consider sociodemographic effects. Mean total scores were, as with previous South African samples, below the established cut-off point. This cut-off point identified a majority of NCH participants with possible MCI, similar to the figures observed in a comparable South African sample (Mienie, 2020), and challenged the universal use of ≤ 26 as cut-off point.

Ceiling effects were observed on a number of items, due to, among others, the general level of education and good health of the sample. Ceiling effects would be appropriate in healthy populations where good performance would be expected and desired. Comparatively poorer performance was observed on three items, namely the second repeat sentence, the second abstraction item and the delayed recall of ‘daisy’. All three were previously reported in South African samples (Mienie, 2020; Robbins et al., 2013). The clinical notes of the administering psychologists’ records attributed poorer performance on the second repeat sentence to possible cadence or grammar complexity, which would challenge non-English-as-first-language speakers. In the case of ‘daisy’, many participants were not familiar with the word or its meaning, and the cue of ‘it is a flower’ did therefore not aid their recall.

Very few participants indicated that they could not name any of the animals. Where no points were awarded, it was not because no name had been offered, but rather because of incorrect naming. The animals were originally selected because of supposed low familiarity. However, ‘lion’ is well known in South Africa, which likely contributed to its ceiling effect. Rhinoceros is also indigenous to South Africa, but was often spontaneously named as hippopotamus or buffalo (similar to the observations of Mienie, 2020, and Robbins et al., 2013).

Previous South African reports on the association of decreasing total scores with advancing age were supported, with comparable effect sizes (Beath et al., 2018; Kirkbride et al., 2022). Similarly, the general lack of gender differences followed the established literature and supports previous South Africa reports (Kirkbride et al., 2022). The only significant gender difference was in visuospatial performance, where men scored higher. Many men in the sample had engineering or technical backgrounds and often reported technical drawing as subject during training, which could have influenced their performance on, for example, the cube copy task. More work is necessary to understand the influence of specific work experience on performance.

While the mean difference between the two language groups was only about half a point, test outcome was biased against not-English-as-first-language speakers. The high level of education in the sample likely contributed to the small difference. The language domain accounted for most of the difference, aided by the memory domain (which was tested in the verbal modality). Unfamiliar words, such as daisy, that had no meaning were more difficult to recall.

English-as-first-language speakers produced on average one word more on the phonetic fluency task, but the wide variability across individuals within each general language group precludes easy interpretation. A substantial number of participants, across the language groups, could produce 10 words and only narrowly missed the ≥ 11 cut-off for earning a point. Disparate backgrounds in terms of quality of education (not measured in this sample) may have contributed to the wide variability within each of the two language groups.

It did appear that neither self-reported English proficiency nor additional vocational training (in English), nor using English in workplace, was enough to offset the benefit from English as language of upbringing and daily home use to MoCA performance.

The second objective was to extend the psychometric analysis to consider indices of structural validity. Confirmatory factor analysis outcomes supported the reported tendency of MoCA items to converge towards a multidimensional structure – that is, reflecting neurocognitive domains – that correlated to a general factor (Freitas et al., 2012b), which in turn suggests that the total score is indeed a measure of global cognitive functioning (Sala et al., 2020). This was the first South African study to report on the specific techniques used to examine dimensionality, and it will thus need to be replicated to confirm the results.

Measurement invariance for gender has previously been reported (Sala et al., 2020), and such metric invariance was also observed in this sample of South African workers. Further, metric invariance for language was also found. This was against the significant though small difference in mean scores between the language groups and may suggest that language background, rather than item or scale structure, contributed to the difference in mean total scores. This study was the first to test measurement invariance in a South African sample, and this will need to be repeated in samples with greater diversity of English exposure, to clarify the role of language proficiency bias on scale responses.

Internal consistency values suggested low reliability, but this may be an artefact of the sample, where too many items presented with ceiling effects. This was the first South African study to report McDonald’s ω, and this statistic is recommended for use in future studies (Dunn et al., 2014), particularly given that the multidimensionality of the MoCA has now been repeatedly described, and Cronbach’s α would not be an appropriate metric.

The third objective was to consider discriminant validity for MCI and further to illustrate the usefulness of developing grey-zone lower and upper thresholds. The MoCA significantly and substantially discriminated between the NCH and MND samples with similar age and education, with clinically useful AUC observed, supporting the findings of Beath et al. (2018). Optimal sensitivity and specificity were found at ≤ 23. As with all previous South African studies, mean total scores for this NCH sample were below the established cut-off point of 26.

It is not clear whether the sensitivity and specificity found in this study are sufficiently useful for practical implementation in clinical service. This, together with the potential of intra-individual and situation-specific conditions influencing test performance, may make the development of grey-zone scores worth considering for future application. In this study, the small sample size is a recognised limitation, and the use of grey-zone scores is presented here as illustration only. The availability of upper and lower threshold scores may aid decision-making. For example, a score below the lower threshold (< 21 in this sample) could indicate the need for urgent action, while a score below the upper threshold (< 24 in this sample) could indicate an ‘at-risk’ person who may need to be monitored closely. Large sample studies would be required to develop actual threshold cut-off points that could be used in primary healthcare settings where specialised expertise is not readily available.

It has become clear that the use of ≤ 26 as universal cut-off point independent of context can no longer be defended, not even for skilled workers with English as first language. Neurocognitive test performance is context-specific, with local cultural and language backgrounds influencing the completion of screening tools, such as the MoCA (Cockcroft, 2020). Within the South African context, a number of items may need to be modified before local validation can be attempted (Beath et al., 2018; Robbins et al., 2013). For example, animal naming may need to use region-specific stimuli in the form of animals with lower familiarity, but whose names are in common use. Phonetic fluency may require a different stimulus letter option, depending on the language of the respondent, or even a lower threshold for people who do not have English as first language (e.g. 10 words rather than 11). The repeat sentence task, particularly the second sentence, may need modification that takes into account grammar complexity, syllable count and its associated cadence, factors that again are specific to the language of the respondent. The memory task needs to update the stimulus items to include words with higher familiarity; the same would apply to the second abstraction task. The use of the clock task may in a generation or two become problematic, as more and more people may not be familiar with an analogue-time clock face. Lastly, while currently still controversial, there is a debate regarding whether the increased prevalence of social media use is beneficial (Quinn, 2018) or detrimental (Sharifian & Zahodne, 2020) to memory performance in older adults. Possible changes in memory performance could thus in future require a reconsideration of how screening for memory is factored into contemporary scoring systems.

### Limitations

This was a convenience sample and would not necessarily represent the larger South African population. Further, the clinical sample was also small, and findings based on their data should be for illustrative use rather than final conclusions. English proficiency was not objectively tested, but assumed, based on self-report, level of education and workplace language use. This is, however, in line with clinical practice. The study used cases from two assessors, with the associated risk of administration biases (Society for Industrial and Organizational Psychology, 2018). To mitigate the risk, the two psychologists met two-weekly, to align administration and scoring processes. Further, to reduce bias, MoCA now requires users to be certified, and this is recommended for future use of the MoCA.

## Conclusion

This study extended previous research on South African samples, focussing on NCH working adults with good English proficiency. It replicated local findings of mean total scores falling below established cut-off points. Analysis further indicated a multidimensional structure of cognitive domains converging on a general factor that reflects global cognitive functioning. Measurement invariance for gender and language was also confirmed.

In this sample, the total MoCA score could distinguish between NCH and MND samples, with optimal sensitivity and specificity around ≤ 23. The potential for establishing grey-zone thresholds to account for contextual factors influencing performance was thus proposed.

Overall, the study highlighted the need for context-specific adaptation in cognitive assessments, especially for non-English-as-first language speakers, to enhance their practical utility. It further challenged the universal use of ≤ 26 as cut-off for cognitive impairment in South Africa. In the ever-evolving landscape of cognitive screening, tailored approaches are vital for accurate evaluations and improved healthcare outcomes.
